# Mechanical Surgery

**Published:** 1898-08

**Authors:** 


### MECHANICAL SURGERY.

According to the *Physician and Surgeon* for August, an electric circular saw now renders service in lieu of the knife in all amputations performed at the Boston Emergency Hospital. Recourse to this agency obviates the necessity of an anæsthetic, as the rapidity of its action renders the operation painless, and greatly decreases shock. Ligatures are not required, and the house surgeon says that patients treated by this method fare better than those subjected to the older procedure.

When, we may ask, is some ingenious surgeon mechanic going to give us an amputating machine into which the patient can be put at one end, and come out at the other with his limb amputated, the arteries ligated, the flaps sutured, and the dressings applied—as they say hogs are turned into sausage in Chicago?—*New York Medical Journal.*

